# Validation Including Uncertainty Estimation of a GC–MS/MS Method for Determination of Selected Halogenated Priority Substances in Fish Using Rapid and Efficient Lipid Removing Sample Preparation

**DOI:** 10.3390/foods8030101

**Published:** 2019-03-18

**Authors:** Slávka Nagyová, Peter Tölgyessy

**Affiliations:** Water Research Institute, Slovak National Water Reference Laboratory, Nábrežie arm. gen. L. Svobodu 5, 812 49 Bratislava, Slovak Republic; slavka.nagyova@vuvh.sk

**Keywords:** QuEChERS, dispersive liquid–liquid microextraction, sulfuric acid treatment, gas chromatography, tandem mass spectrometry, priority substances, fish samples

## Abstract

A rapid method is proposed for the determination of selected H_2_SO_4_ stable organic compounds—eight organochlorines (OCs; hexachloro-1,3-butadiene, pentachlorobenzene, hexachlorobenzene, hexachlorocyclohexane—HCH—isomers, heptachlor) and six polybrominated diphenyl ethers (PBDEs; BDE-28, 47, 99, 100, 153, 154)—in fish samples. In the method, a modified QuEChERS (quick, easy, cheap, effective, rugged and safe) sample preparation using pH-tuned dispersive liquid–liquid microextraction (DLLME) and H_2_SO_4_ digestion fish extract clean-up is followed by gas chromatography–triple quadrupole tandem mass spectrometry (GC–QqQ-MS/MS) analysis. The method was validated in terms of linearity, limits of the method, recovery, accuracy, analysis of standard reference material (NIST SRM 1946), and estimation of combined uncertainty of the measurement (top-down approach). For validation, chub composite samples were used, and subsequently, the method was successfully applied to analysis of real samples of eight fish species. Finally, the method passed the analytical Eco-Scale evaluation as “an acceptable green analysis method”, and showed its advantages (simplicity, rapidity, low cost, high extract clean-up efficiency, good sensitivity) when compared to other reported QuEChERS based methods.

## 1. Introduction

Anthropogenic halogenated organic compounds synthesized as pesticides, solvents or fire retardants have been found to pose a serious threat to aquatic environments, wildlife and humans due to their toxic, persistent and bioaccumulative properties [1]. Because of these harmful effects and impacts, the production and use of large number of organochlorine (OC) pesticides and certain brominated flame retardants was banned or severely restricted in the European Union (EU), and other parts of the world [2], but their presence and release in the environment can be expected over the next decades. 

One of the ways human health can be endangered by these substances is through consuming fish living in contaminated waters and accumulating the toxic chemicals in their tissues. Therefore, it is necessary to monitor and analyze fish contamination to protect humans from the consumption of contaminated food. The regulatory limit applicable to residues of pesticides in fish and fishery products is the default maximum residue level (MRL) of 10 µg/kg set by the EU in Regulation 396/2005, which concerns public health and is relevant to the functioning of the internal market [3]. For the determination of PBDEs in fish and other seafood, the EU Commission recommends (recommendation 2014/118/EU) use of analytical methods with a limit of quantification of 0.01 µg/kg wet weight or lower [4]. 

This paper is focused on the determination of eight OC compounds (hexachloro-1,3-butadiene, pentachlorobenzene, hexachlorobenzene, hexachlorocyclohexane—HCH—isomers, heptachlor) and six polybrominated diphenyl ethers (PBDEs; BDE-28, 47, 99, 100, 153, 154) in fish that were selected from the EU list of priority substances in the field of water policy [5] and from the U.S. EPA (Environmental Protection Agency) priority pollutants list [6]. A great variety of extraction techniques have been applied in the analysis of organic halogenated compounds in fish samples. Among others, solid–liquid extraction (SLE), Soxhlet extraction, accelerated solvent extraction (ASE), supercritical fluid extraction (SFE), microwave-assisted extraction (MAE), matrix solid-phase dispersion (MSPD), and so-called QuEChERS method have been reported in literature [7,8,9,10]. The presented methods include both traditional extraction methods (SLE, Soxhlet) which are quite laborious, time-consuming (extraction duration up to 24 h), and require large amounts of organic solvents (up to few hundreds of mL), and novel methods with shortened extraction times (to 10–60 min), reduced solvent consumption, often with reduced cost, and that are amenable to automation. However, the disadvantage of ASE, SFE, and MAE methods lies in the cost of equipment setup.

In the last few decades, the QuEChERS method with the advantages summarized in its acronym (quick, easy, cheap, effective, rugged, and safe) has become a very attractive sample preparation method in food analysis [11]. Overall, this procedure consists of two main parts: extraction with a solvent (mostly acetonitrile, MeCN) and partitioning salts (MgSO_4_, NaCl), and extract clean-up using dispersive solid-phase extraction (dSPE) technique. However, the dSPE clean-up is not fully sufficient for the analysis of high fat matrices and, therefore, the clean-up part of the original QuEChERS method has gone through various modifications to enhance the co-extractives (mainly lipid) removal efficiency (use of freezing, dual dSPE, gel permeation chromatography, silica minicolumn, EMR-lipid sorbent) [9,12,13,14,15,16,17].

Currently, a novel method for clean-up of fatty MeCN extracts (after QuEChERS extraction), which is suitable for determination of H_2_SO_4_ stable organic compounds in complex biological samples, was developed in our laboratory [18]. The sample extract clean-up combines the pH-tuned dispersive liquid–liquid microextraction (DLLME) with conc. H_2_SO_4_ digestion. This clean-up offers many advantages, including high lipid removal efficiency, rapidity, analyte enrichment without evaporating solvent, low cost (cheap chemicals, no need for expensive sorbents), low chemicals and glassware usage, no need of special laboratory equipment, and less bench space. The lipid removal involves complete removal of fatty acids, which are partitioned from the organic phase into the alkaline aqueous phase in the DLLME clean-up step [18]. The disadvantage is the use of toxic and hazardous chemicals (CHCl_3_, hexane, MeCN, H_2_SO_4_), however, they are applied in small quantities.

The aim of this study was the validation including uncertainty estimation of a rapid and non-laborious method for determination of selected H_2_SO_4_ stable halogenated priority substances in fish employing modified QuEChERS sample preparation followed by gas chromatographic and triple quadrupole tandem mass spectrometric (GC–QqQ-MS/MS) analysis.

## 2. Materials and Methods

### 2.1. Standards and Reagents

Neat standards of pentachlorobenzene, hexachlorobenzene, *alpha*-HCH, *beta*-HCH, *delta*-HCH and heptachlor (purity: 98.1–99.5%) were obtained from Dr. Ehrenstorfer (Augsburg, Germany). Neat standards of hexachloro-1,3-butadiene (96%) and lindane (97%) were from Sigma–Aldrich (Steinheim, Germany). Standard of 2,4,5,6-tetrachloro-*m*-xylene (99.0%) in cyclohexane at 10 µg/mL was prepared by Dr. Ehrenstorfer. Individual PBDE standards BDE-28, BDE-47, BDE-77, BDE-99, BDE-100, BDE-153 and BDE-154, each at 50 µg/mL in nonane (≥98%), were produced by Cambridge Isotope Laboratories (CIL, Andover, MA, USA).

Anhydrous magnesium sulfate, sulfuric acid, acetone, chloroform and toluene, all Emsure grade, cyclohexane and ethyl acetate (SupraSolv), and n-hexane (UniSolv), were purchased from Merck (Darmstadt, Germany). Sodium chloride, anhydrous sodium acetate (both ReagentPlus) and MeCN (Chromasolv) were obtained from Sigma–Aldrich.

Sodium acetate solution at 0.5 M was prepared by dissolution of CH_3_COONa in Milli-Q water produced by a Direct-Q 3 water purification system (Millipore, Molsheim, France). Stock solutions of each OC compound obtained as a neat material were prepared in cyclohexane at a concentration of 5 mg/mL, with the exception of a solution of *beta*-HCH, which was prepared in a mixture of cyclohexane and acetone (4:1, *v*/*v*) at a concentration of 1 mg/mL. Standard working mixtures of eight OC compounds were prepared by dilution of their stock solutions with cyclohexane to obtain concentrations of 1 and 10 µg/mL. An internal standard (IS) solution of 2,4,5,6-tetrachloro-*m*-xylene at 1 µg/mL was prepared by dilution of the stock standard solution with cyclohexane. Standard working mixtures of six PBDEs (BDE-28, 47, 99, 100, 153 and 154) at concentrations of 5 and 0.5 µg/mL were obtained by dilution of the individual standard solutions with toluene. An IS solution of BDE-77 at 5 µg/mL was also prepared from the stock standard solution by dilution with toluene. 

### 2.2. Fish Samples

The proposed method was validated and verified using samples of nine different fish species: European chub (*Squalius cephalus*), crucian carp (*Carassius carassius*), European perch (*Perca fluviatilis*), northern pike (*Esox lucius*), zander (*Sander lucioperca*), brown trout (*Salmo trutta*), Atlantic salmon (*Salmo salar*), Alaska pollock (*Theragra chalcogramma*), and lake trout (*Salvelinus namaycush*). The first six species were collected by electrofishing during a fish survey performed in Slovak water bodies in 2015 within the project: Monitoring and assessment of water body status (see Funding). The samples of salmon and pollock were purchased as frozen skinless fillets from a local supermarket. The samples were prepared as composite homogenates from several pieces (from 2 to 7) of the whole fish (chub, perch, pike, and trout) or homogenates from single fish (chub and remaining species). The samples were homogenized using a knife mill Grindomix GM 200 (Retsch, Haan, Germany) to give a wet weight of about 600 g and were stored in a freezer at −20 °C until extraction and analysis. The main part of the study was done using chub composite samples.

Accuracy of the method was demonstrated by the analysis of the standard reference material SRM 1946 (Lake Superior Fish Tissue) which was prepared from lake trout by the National Institute of Standards and Technology (NIST, Gaithersburg, MD, USA). This SRM was a frozen fish tissue homogenate with 10.2% of extractable fat and 71.4% of water.

### 2.3. Lipid and Moisture Determination

The lipid and moisture content of fish homogenate samples was determined by gravimetric methods according to our work [19]. For total lipid determination, 5 g of fish homogenate was extracted with 5 mL of acetone/ethyl acetate solvent mixture (6:4, *v*/*v*) by shaking with a vortex mixer (Stuart SA8, Bibby Scientific, Stone, UK) for 3 min and, after addition of 2 g of MgSO_4_ and 0.5 g NaCl and shaking for 3 min, the organic phase was separated by centrifugation (centrifuge Rotina 380, Hettich, Tuttlingen, Germany). An aliquot of the organic phase was dried to constant weight at 103 °C, and the percent lipid content was calculated from the mass of the final residue. The moisture content was determined from the mass difference of 2–3 g portions of fish homogenate before and after a 24 h drying at 60 °C. For all fish sample homogenates, the lipid content was determined in triplicates (results in the range 0.63–16%) and the moisture content in duplicates (58–81%).

### 2.4. Sample Preparation

An aliquot of 5 g of fish homogenate was weighed into a 50-mL polypropylene centrifuge tube (Corning, CentriStar, Sigma–Aldrich, Steinheim, Germany) and spiked with IS solutions and mixture of analytes (in case of standard addition). After 15 min, the spiked homogenate was mixed with 5 mL of MeCN and shaken by a vortex mixer at 800 rpm for 1 min. Then, a salt mixture of 2 g of anhydrous MgSO_4_ and 0.5 g of NaCl was added, and again, the tube was shaken vigorously for 1 min. Next, the sample was centrifuged at 5000 rpm for 5 min.

In the DLLME step, a 1-mL aliquot of supernatant was transferred to a 15-mL centrifuge tube with 4 mL of 0.5 M CH_3_COONa solution. Then, 50 µL of CHCl_3_ was injected rapidly into the mixture; the tube was vortexed for 1 min and centrifuged at 5000 rpm for 5 min.

Finally, for the H_2_SO_4_ clean-up step, the whole sedimented phase was placed in a 1.7-mL clickseal microcetrifuge tube (GoldenGate Bioscience, Claremont, CA, USA) and 1 mL of concentrated H_2_SO_4_ was added slowly. The tube was sealed, shortly shaken by hand and then 80 µL of hexane was added to the top of the solution. After short shaking, the tube was centrifuged in a microcentrifuge (Mikro 220R, Hettich) at 10,000 rpm for 5 min. The upper phase was transferred into a GC vial equipped with a 100-µL glass insert and was then ready for GC–MS/MS analysis.

### 2.5. Instrumental Analysis

Analyses were performed using an Agilent 7890B GC combined with a 7000D QqQ-MS/MS system (Agilent Technologies, Wilmington, DE, USA). The GC was equipped with a multimode inlet and for the injection of sample extracts a multipurpose sampler (MPS) from Gerstel (Mülheim a/d Ruhr, Germany) was used. Two identical Agilent HP-5MS UI capillary columns (15 m × 0.25 mm I.D., 0.25 µm film thickness) connected in series (via Agilent Purged Ultimate Union) were used for separation of the analytes, while a deactivated fused-silica tube (1 m × 0.32 mm I.D.) was used as a precolumn. Helium was used as the carrier gas at constant flow rates of 1.1 and 1.3 mL/min for the first and the second column, respectively.

Sample injection (1 µL) was carried in splitless mode (1 min) at 275 °C. The oven temperature was programmed from 60 °C (1 min hold) to 170 °C at a rate of 40 °C/min, and then to 300 °C (1.75 min hold) at a rate of 10 °C/min. After each run, a 3 min column clean-up was performed employing a mid-column backflush. The backflush was conducted at 305 °C, by applying helium to purged ultimate union at 320 kPa. This program resulted in a total run time of 21.5 min.

The mass selective detector (MSD) was operated using electron ionization at 70 eV in the multiple reaction monitoring (MRM) mode. The retention times (*t_R_*), quantifier and qualifier transitions for the selected analytes are listed in Table 1. Dwell times were in all cases set at 10 ms. The MSD transfer line was at 280 °C, ion source at 300 °C, and quadrupoles at 150 °C. The QqQ collision gas was nitrogen at 1.5 mL/min, and quench gas was helium at 2.25 mL/min. Agilent MassHunter software was used for instrument control and data analysis.

The quantification process was performed using a single point standard addition method applying Equation (1):

(1) $$c_{i}=c_{ad}\times \frac{A_{i}/A_{IS}}{\left(A_{i+ad}/A_{ISsa}\right)-\left(A_{i}/A_{IS}\right)}$$

where *c_i_* is the determined analyte concentration, *c_ad_* is the added concentration to the sample, *A_i_* and *A_IS_* are the peak areas of the analyte and IS from the unknown sample analysis, *A_i+ad_* and *A_ISsa_* are the peak areas of the analyte and IS from the analysis with standard addition. For this purpose, the concentration of each added analyte and of IS tetrachloro-*m*-xylene was 10 µg/kg and of IS BDE-77 was 20 µg/kg. This was appropriate for the studied range and in agreement with the study of Frenich et al. [20]. In the whole work, the concentrations of the analytes are presented on a wet weight basis.

### 2.6. Matrix Effect Evaluation

The evaluation of the matrix effect (*ME*) on the GC–MS/MS analysis was based on comparing the analyte response measured in matrix-matched extracts spiked after QuEChERS extraction and processed by DLLME and H_2_SO_4_ clean-up procedure and the response measured in a corresponding neat solvent solution. The *ME* was calculated from replicate analyses as the average percent suppression or enhancement in the peak area using the following Equation (2):

(2) $$ME(\%)=\frac{Peak\;area\;in\;matrix\;matched\;standard-Peak\;area\;in\text{ ​}solvent\;standard}{Peak\;area\;in\;matrix\;matched\;standard}\times 100$$

A positive value of *ME* corresponds to a matrix-induced enhancement of analyte response, whereas a negative value corresponds to a suppression effect. 

### 2.7. Measurement Uncertainty Calculation

The combined measurement uncertainty was estimated according to the top-down approach using quality control (QC) charts, validation data and the uncertainty of purity of analytical standards [21,22]. The random error contribution to the measurement uncertainty was characterized by the within-lab (intermediate) reproducibility (*u_r,repro_*), which was calculated as relative standard deviation (RSD%) from at least 20 independent consecutive measurement values taken from the QC charts (QC samples spiked at 5 µg/kg). 

Systematic components of uncertainty were characterized as the relative bias (*B_r_*) and the uncertainty of the systematic error (*u_r,cm_*) and were determined by measuring QC samples at conditions of repeatability. The *B_r_* was quantified using the Equation (3):

(3) $$B_{r}(\%)=\frac{c_{m}-c_{ref}}{c_{ref}}\times 100$$

where *c_m_* and *c_ref_* are the mean measured concentration and reference concentration of the studied analyte, respectively. For determination of *u_r,cm_*, the QC samples were analyzed with minimum number of replicates of 10. For calculation, the following Equations (4) and (5) were used:

(4) $$u_{cm}=\frac{SD}{\sqrt{n}}$$

(5) $$u_{r,cm}(\%)=\frac{u_{cm}}{c_{ref}}\times 100$$

where *u_cm_* is the standard uncertainty of *c_m_*, *SD* is the standard deviation, and *n* is the number of replicates.

The uncertainty of purity of analytical standards (*u_r,ref_*) was determined by dividing the expanded combined uncertainty (*U_r,ref_*) (given in manufacturer’s certificate) by the coverage factor *k* = 2, or was estimated on the basis of Equation (6) derived from rectangular distribution (in case of absence of the certificate):

(6) $$u_{r,ref}(\%)=\frac{0.5\times \left(100-y\right)}{\sqrt{3}}$$

where *y* (%) represents the purity of standard given in the manufacturer’s specification.

All the characterized uncertainty components were combined by the error propagation rule to obtain the relative combined measurement uncertainty (*u_r,tot_*) using Equation (7):

(7) $$u_{r,tot}(\%)=\sqrt{u_{r,repro}^{2}+B_{r}^{2}+u_{r,cm}^{2}+u_{r,ref}^{2}}$$

Finally, the expanded combined uncertainty (*U_r,tot_*) was calculated by multiplying the *u_r,tot_* with the coverage factor of 2 (95% confidence level).

## 3. Results and Discussion

### 3.1. Instrumental Analysis

The instrumental analysis conditions summarized in Section 2.5 were selected based on our previous studies [9,18]. In contrast to study [18], in which sample loading for GC analysis was carried out by thermal desorption of fish extract from the insert placed in the thermal desorption tube, a liquid injection of sample extract was employed.

### 3.2. ME

The QuEChERS extracts from the chub homogenate sample with lipid content of 5.2% and water content of 74.6% spiked with test analytes at concentration level of 5 ng/mL and treated by DLLME and H_2_SO_4_ were used for *ME* evaluation according to 2.6. The *ME*s were calculated from five replicate analyses. The obtained *ME* values in the range from −5.1% to 10.5% (see Table 2) show very low enhancement or suppression of chromatographic response of the studied analytes. For comparison, *ME*s presented in the studies employing modified QuEChERS methods with dSPE clean-up were for selected OC pesticides incomparably higher. The *ME* values, in the studies [14] and [23], were 32 and 175.7% for *beta*-HCH, and 63 and 219.4% for *delta*-HCH, respectively. The *ME*s for *alpha*-HCH, hexachlorobenzene, lindane and heptachlor were in the study [23] calculated as 40.3, 27.4, 34.7 and 28.8%, respectively. For seven PBDEs, Sapozhnikova and Lehotay [24] observed matrix-induced suppression of chromatographic response with *ME* values in the range from −16% to −26% when using unbuffered QuEChERS method with dSPE clean-up for fish sample preparation. The low *ME*s obtained by the proposed method in this study indicate the high efficiency of fish extract clean-up.

### 3.3. Method Validation

Within-laboratory validation of the proposed method was carried out using two chub composite samples with lipid and water content of 1.9% and 5.2%, and 80% and 75%, respectively, with absence or low levels of the analytes of interest. The validation was performed in terms of linearity, limits of detection (LOD), limits of quantification (LOQ), recovery, accuracy involving evaluation of precision and trueness and analysis of SRM and, finally, the combined uncertainty of the measurement was estimated.

#### 3.3.1. Linearity

Response linearity was assessed by studying calibration curves from the analyses of matrix-matched standards of the test analytes. The standards were prepared by spiking the extract of chub composite sample (lipid content of 1.9%) with standard working mixtures to obtain seven concentration levels (0.1, 0.5, 1, 5, 15, 30 and 60 µg/kg). The response linearity was evaluated on the basis of coefficients of determination (*R*^2^) and RSDs of the relative response factors (RRF). The RRFs of the analytes were calculated relative to the internal standard at each concentration level applying a blank correction. In Table 3 it can be seen that the obtained calibration functions were linear for all the analytes, with *R*^2^ values above 0.999 and RSDs of the RRFs in the range of 5.6–13%.

#### 3.3.2. Limits of the Method

**Figure 1:**
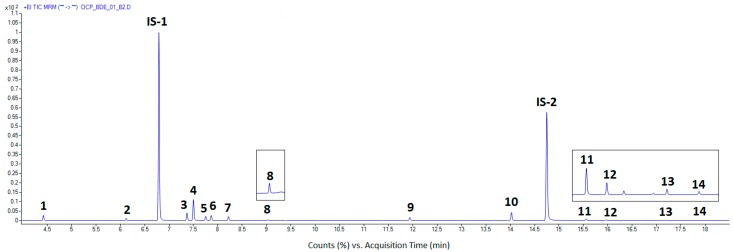
Total ion MRM chromatogram from the GC–QqQ-MS/MS analysis of the blank chub composite sample spiked with the test analytes at LOQ level of 0.1 µg/kg, and internal standards at 10 (IS-1) and 20 µg/kg (IS-2), respectively. Peaks: 1—hexachloro-1,3-butadiene, 2—pentachlorobenzene, IS-1—tetrachloro-*m*-xylene, 3—*alpha*-HCH, 4—hexachlorobenzene, 5—*beta*-HCH, 6—lindane, 7—*delta*-HCH, 8—heptachlor, 9—BDE-28, 10—BDE-47, IS-2—BDE-77, 11—BDE-100, 12—BDE-99, 13—BDE-154, 14—BDE-153. Hexachlorobenzene (at 0.64 µg/kg), BDE-47 (0.35 µg/kg), BDE-100 (0.18 µg/kg), BDE-154 (0.23 µg/kg), and BDE-153 (0.15 µg/kg) were present in the sample before spiking.

Ten replicate analysis of the blank chub composite sample spiked at 0.1 µg/kg were used for determination of the limits of the method. The LODs and LOQs were calculated as three and ten times the standard deviations (SD) of the results, respectively. As can be seen in Table 3, the LOQs for the test analytes were in the range from 0.07 to 0.17 µg/kg, much lower than the default MRL of 10 µg/kg applicable to pesticides in fish, and approaching the EU Commission recommended value of 0.01 µg/kg for LOQ of analytical methods for the determination of PBDEs in fish and other seafood [4]. In the published studies [14,23] employing similar GC–MS/MS instrumentation and applying the QuEChERS methodology with dSPE clean-up for determination of the test OC compounds in fish, the LOQs were in the ranges 2–13 µg/kg, and 1–5 µg/kg, respectively. In these studies, unlike with the employed SD calculation approach, a less/not appropriate (for QqQ-MS/MS detection, [22]) method based on signal to noise (S/N) estimation was used for LOQ evaluation, and therefore the obtained LOQs are hardly comparable. The lowest calibration levels (LCLs) of the test PBDEs obtained in the study [24] using original QuEChERS sample preparation and GC–MS/MS method were in the range 0.5–5 µg/kg. For illustration, Figure 1 shows a total MRM chromatogram from the analysis of the blank chub composite sample spiked with the test analytes at LOQ level of 0.1 µg/kg. It can be seen the baseline separation of all the analytes with no interferences from the matrix.

#### 3.3.3. Recovery

Recovery experiments were conducted with the chub composite sample (lipid content of 1.9%) spiked with the test analytes at levels of 1, 5, 15, 30 and 60 µg/kg, respectively, covering the linearity range of the method. A single-point standard addition method (see Section 2.5) was used for quantification, which enabled us to solve the problem of absence of suitable fish matrix free of analytes of interest that is necessary for matrix-matched calibration at low concentration levels, and also helped to overcome the negative effects of matrix components. From Table 4 it can be seen that the obtained recoveries are in the range of 57%–124% with RSDs in the range of 2%–18%. These results are acceptable according to the requirements of the EU guidance document SANTE/11813/2017 for pesticide residues analysis in food, because the recoveries outside the range of 70%–120% are consistent (RSDs ≤ 20%) and are not lower than 30% or above 140% [25]. 

#### 3.3.4. Accuracy

The accuracy of the method was studied in terms of two components—precision and trueness [26]. The precision was evaluated as intra-day (PRE_intra_) and inter-day (PRE_inter_) precision and expressed by RSD for the repeated analyses of QC samples (lipid content of 5.2%) spiked with the test analytes at 5 µg/kg. The PRE_intra_ was determined by the analysis of ten replicates QC samples on one day, while PRE_inter_ was calculated from measurements of four replicates QC samples per day analyzed on four consecutive days. The trueness of the method was evaluated on the basis of ten measurements from the determination of PRE_intra_ and expressed as a mean recovery (*R*) and a mean relative bias (*B_r_*). The results from the accuracy assessment are presented in Table 3. It can be seen that RSDs for PRE_inta_ and PRE_inter_ are in the ranges of 0.5%–11% and 3.2%–16%, respectively, showing a satisfactory precision of the method. The good trueness of the method is demonstrated by the values of Rec and *B_r_* in the ranges of 87%–107% and −13.0%–7.4%, respectively.

Finally, the method’s accuracy was studied by the analysis of the NIST SRM 1946 standard fish tissue reference material prepared from lake trout. Table 5 presents results obtained for those test analytes for which certified concentrations were available. According to obtained trueness and precision, acceptable results (trueness in the range 70%–120%, RSD ≤ 20%) were obtained for eight from nine analytes (except BDE-28). However, when comparing with the certified ranges, three results (lindane, BDE-28 and BDE-99) were outside and one result (BDE-154) was at the border of the certified range.

#### 3.3.5. Uncertainty of Measurement

To evaluate the uncertainty of measurement, a top-down approach has been used that utilizes data from validation and QC charts and is much simpler than the GUM (Generalized Uncertainty Method) bottom-up approach [27]. The combined measurement uncertainty was estimated according to Section 2.7 and the resulting values together with the individual uncertainty components are summarized in Table 6. As can be seen in Table 6, generally the most significant contribution to the measurement uncertainty was associated with the random error characterized by the within-lab reproducibility (*u_r,repro_*). In several cases, the highest uncertainty component was the relative bias (*B_r_*) representing the systematic error of the measurement. The *B_r_* values were in the broadest range among the evaluated uncertainty components from 0.2 to –13.0%. The resulting values of the expanded combined uncertainty (*U_r,tot_*) for the test analytes were in the range between 14.4 and 28.7%, being in accordance with the requirement (50%) of the EU guidance document SANTE/11813/2017 [25]. For comparison, in the study combining the QuEChERS method with GC–MS analysis for the determination of OC compounds in fish, Olivares et al. [28] estimated the combined measurement uncertainty for hexachlorobenzene and lindane at levels of 29.9 and 20.0%, respectively. In the work dealing with determination of OC pesticides in meat employing ASE, mini-silica column purification and GC–ECD analysis, Dimitrova et al. [29] obtained for hexachlorobenzene and HCH isomers expanded uncertainties in the range of 14.6%–17.9%. In the currently published study [30] concerning the determination of halogenated flame retardants by GC–API-MS/MS and GC–EI-MS after ASE and multi column clean-up, the values of expanded measurement uncertainties for the PBDEs of our interest in fish fillet were below 50%.

### 3.4. Application of the Method to Real Samples

The applicability of the proposed method was evaluated by extraction and determination of the test analytes in homogenate samples of eight different fish species listed in Table 7. The lipid content of the analyzed fish (see Table 7) was in the range from 0.63% to 16% and the moisture content in the ranged from 58% to 81%, respectively. Table 7 presents the determined concentrations of the test analytes in fish homogenates and the relative recoveries (*RR*) of the analytes determined after their addition to the sample at concentration of 10 µg/kg. In general, the fish with the highest lipid content were the most contaminated (trout, salmon), while the least contaminated were those with the lowest lipid content (crucian carp, pollock). For all samples, hexachlorobenzene and BDE-47 were the most frequently detected analytes as well as the ones present at the highest concentrations. The *RR* values for the test analytes were in the range of 53%–128% with RSDs in the range of 1%–23%.

**Figure 2:**
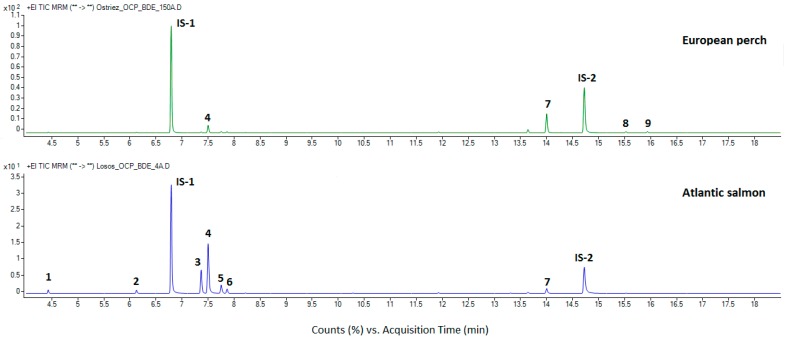
Total ion MRM chromatograms from the GC–QqQ-MS/MS analysis of extracts prepared from samples of European perch and Atlantic salmon. Peaks: 1—hexachloro-1,3-butadiene, 2—pentachlorobenzene, IS-1—tetrachloro-*m*-xylene, 3—*alpha*-HCH, 4—hexachlorobenzene, 5—beta-HCH, 6—lindane, 7—BDE-47, IS-2—BDE-77, 8—BDE-100, 9—BDE-99.

**Figure 3:**
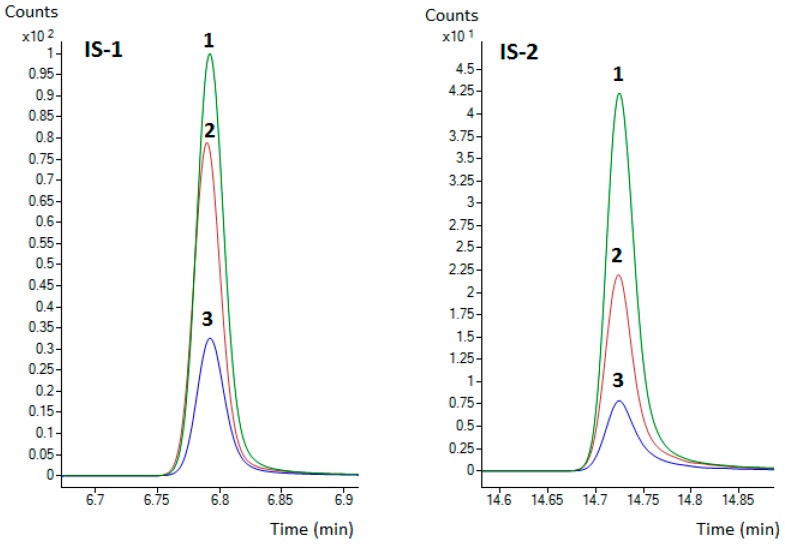
Dependence of the IS peak size on the lipid content of analyzed fish matrix. 1—perch (3.0%), 2—trout (8.2%), 3—salmon (16%). Other parameters of these fish samples are presented in Table 7.

In Figure 2, chromatograms from the analysis of low fat (perch, 3.0%) and high fat (salmon, 16%) fish samples are shown. In both total MRM chromatograms a high selectivity for the detected analytes and absence of matrix components can be observed. A difference between the counts obtained for ISs in the first and second chromatograms can be seen that can be related to the effect of the sample lipid content on the analysis. This is demonstrated in Figure 3, where a dependence of the IS peak size on the lipid content of the analyzed fish is presented. Therefore, the ISs and analytes peak areas generally decreased with the increasing lipid content of the matrix.

### 3.5. Method’s Analytical Eco-Scale Evaluation

The environmental impact of the proposed method was assessed using an analytical Eco-Scale approach that is based on assigning penalty points to parameters of the analytical process (depending on the use of hazardous chemicals, energy consumption, waste generation, etc.) that are not in agreement with the principles of green chemistry [31]. The analytical Eco-Scale analysis results in a score calculated by subtracting the penalty points from a value of 100, which represents an ideal green analysis. The assessment of the proposed method according to criteria set by Gałuszka et al. [31] is presented in Table 8. Due to obtained analytical Eco-Scale total score of 68, the current method can be classified as “an acceptable green analysis method” with low consumption of hazardous solvents.

### 3.6. Comparison of the Proposed Method with Other Reported QuEChERS Based Methods

A comparison of the proposed method with other QuEChERS based methods with enhanced sample extract clean-up for determination of test analytes in fish is presented in Table 9. It can be seen that most of the applied clean-up procedures involve dual dSPE, which in several cases is combined with a freezing-out step for the low temperature lipid precipitation. The use of dSPE requires multiple weighing operations or purchase of custom-made sorbent blends and the freezing-out step significantly prolongs the sample preparation time. In the method used for determination of PBDEs (and other persistent organic pollutants) in salmon fillets [16], the ethyl acetate crude extract was purified applying gel permeation chromatography (GPC) and SPE. This clean-up procedure is rather laborious, requires a GPC instrument and is associated with high solvent consumption (ca. 150 mL per sample). The clean-up procedure described in the present paper is simple, fast, low cost, providing high co-extractives removal efficiency (involves complete removal of fatty acids), but it is only appropriate for the analysis of H_2_SO_4_ stable organic compounds. The LOQs of the presented method belong among the lowest listed in Table 9.

## 4. Conclusions

In this work, a rapid and non-laborious method was proposed for the determination of selected H_2_SO_4_ stable OC compounds and PBDEs in fish samples. The method employing QuEChERS sample preparation with pH-tuned DLLME and H_2_SO_4_ digestion fish extract clean-up followed by GC–QqQ-MS/MS analysis has successfully passed the validation process. The results obtained from the analysis of nine different fish species samples show the applicability of the method for the determination of selected analytes in fish. According to analytical Eco-Scale evaluation, the proposed method can be classified as “an acceptable green analysis method” with low consumption of hazardous solvents. The comparison of the method with other reported QuEChERS based methods shows its advantages such as simplicity, rapidity, low cost, high extract clean-up efficiency and good sensitivity.

## Figures and Tables

**Table 1:** Analytes, retention times and MRM conditions.

Analyte	*t_R_* (min)	MRM Transitions (*m*/*z*)			
		Quantifier	CE (V)	Qualifier	CE (V)
Hexachloro-1,3-butadiene	4.42	225→190	15	260→225	15
Pentachlorobenzene	6.12	248→213	25	250→180	20
Tetrachloro-*m*-xylene (IS-1)	6.79	244→209	15	171→136	15
*alpha*-HCH	7.36	219→183	5	217→181	15
Hexachlorobenzene	7.50	284→214	35	284→249	20
*beta*-HCH	7.75	219→183	5	217→181	15
Lindane	7.86	219→183	5	217→181	15
*delta*-HCH	8.22	219→183	5	217→181	15
Heptachlor	9.02	272→237	25	272→117	35
BDE-28	11.94	246→139	30	406→246	20
BDE-47	14.02	326→217	30	486→326	20
BDE-77 (IS-2)	14.74	326→217	30	486→326	20
BDE-100	15.55	564→404	20	404→297	30
BDE-99	15.97	564→404	20	404→297	30
BDE-154	17.19	644→484	20	484→324	40
BDE-153	17.84	644→484	20	484→324	40

Abbreviations: *t_R_*—retention time; MRM—multiple reaction monitoring; CE—collision energy.

**Table 2:** Matrix effect (*ME*) evaluation for the studied analytes in the spiked QuEChERS extracts after DLLME and H_2_SO_4_ clean-up (*n* = 5).

Analyte	*ME* (%)	RSD (%)
Hexachloro-1,3-butadiene	−5.1	11
Pentachlorobenzene	−1.9	11
Tetrachloro-*m*-xylene (IS-1)	−1.3	12
*alpha*-HCH	−1.2	11
Hexachlorobenzene	3.8	13
*beta*-HCH	9.3	10
Lindane	2.3	10
*delta*-HCH	3.6	11
Heptachlor	1.0	12
BDE-28	1.6	14
BDE-47	5.7	16
BDE-77 (IS-2)	1.3	15
BDE-100	3.9	16
BDE-99	10	14
BDE-154	7.0	14
BDE-153	11	14

**Table 3:** Linearity, limits and accuracy of the proposed method for determination of test analytes in spiked fish matrix.

Analyte	Linear Range (µg/kg)	*R* ^2^	RRF	RRF_RSD (%)	LOD (µg/kg)	LOQ (µg/kg)	Accuracy			
							Precision		Trueness	
							Pre_intra_	Pre_inter_	*R*	*B_r_*
							RSD (%)	RSD (%)	(%)	(%)
Hexachlorobutatadiene	0.1–60	0.99997	1.7	6.7	0.028	0.092	3.0	5.7	95	−4.7
Pentachlorobenzene	0.1–60	0.99975	1.0	5.8	0.036	0.12	2.3	4.9	95	−5.2
*alpha*-HCH	0.1–60	0.99989	3.3	7.1	0.029	0.096	0.5	6.7	89	−11
Hexachlorobenzene	0.1–60	0.99986	1.4	5.9	0.052	0.17	0.8	3.2	107	7.4
*beta*-HCH	0.1–60	0.99973	2.1	11	0.036	0.12	3.4	7.2	88	−12
Lindane	0.1–60	0.99987	2.4	12	0.040	0.13	1.6	7.0	87	−13
*delta*-HCH	0.1–60	0.99982	2.0	12	0.037	0.12	4.7	8.3	91	−9.4
Heptachlor	0.1–60	0.99952	0.46	6.9	0.039	0.13	9.2	16	94	−6.2
BDE-28	0.1–60	0.99984	5.3	5.6	0.037	0.12	8.9	9.0	99	−1.3
BDE-47	0.1–60	0.99994	3.2	13	0.028	0.092	4.8	6.6	102	1.9
BDE-100	0.1–60	0.99984	1.6	10	0.028	0.092	2.9	9.0	100	0.22
BDE-99	0.1–60	0.99986	1.2	5.9	0.021	0.072	6.6	9.2	99	−0.89
BDE-154	0.1–60	0.99939	0.49	12	0.049	0.16	9.0	13	101	1.2
BDE-153	0.1–60	0.99912	0.26	8.1	0.042	0.14	11	13	105	5.1

Abbreviations: *R*^2^—coefficient of determination; RRF—relative response factor; LOD—limit of detection; LOQ—limit of quantification; PRE_intra_—intra-day precision; PRE_inter_—inter-day precision; *R*—recovery; *B_r_*—relative bias.

**Table 4:** Recoveries and RSDs of the test analytes from the spiked chub homogenate.

Analyte	Recovery (RSD) ^a^ (%)				
	1 µg/kg	5 µg/kg	15 µg/kg	30 µg/kg	60 µg/kg
Hexachloro-1,3-butadiene	101 (6)	96 (4)	124 (5)	98 (6)	97 (6)
Pentachlorobenzene	98 (4)	88 (2)	103 (10)	103 (15)	90 (13)
*alpha*-HCH	99 (7)	86 (5)	101 (17)	91 (17)	62 (15)
Hexachlorobenzene	103 (5)	100 (4)	101 (16)	97 (6)	94 (6)
*beta*-HCH	95 (10)	82 (7)	86 (16)	92 (18)	58 (14)
Lindane	94 (10)	88 (5)	96 (16)	90 (18)	59 (15)
*delta*-HCH	91 (10)	85 (16)	89 (16)	88 (18)	57 (14)
Heptachlor	91 (7)	94 (8)	89 (12)	86 (9)	83 (12)
BDE-28	101 (6)	93 (10)	94 (18)	94 (3)	93 (14)
BDE-47	98 (6)	110 (2)	95 (6)	94 (3)	92 (15)
BDE-100	100 (5)	100 (5)	100 (14)	96 (5)	96 (10)
BDE-99	101 (9)	100 (8)	101 (15)	95 (12)	96 (8)
BDE-154	105 (4)	104 (12)	99 (11)	94 (6)	94 (7)
BDE-153	98 (11)	98 (12)	102 (9)	94 (12)	93 (5)

^a^*n* = 5.

**Table 5:** Results from determination of selected chlorinated pesticides and PBDEs in the standard reference material NIST SRM 1946 (Lake Superior Fish Tissue).

Analyte	Certified Value ^a^ (μg/kg)	Determined Value ^a^ (μg/kg)	Trueness (RSD) (%)
Hexachlorobenzene	7.25 ± 0.83	6.47 ± 1.5	89 (2)
*alpha*-HCH	5.72 ± 0.65	5.44 ± 1.4	95 (7)
Lindane	1.14 ± 0.18	0.89 ± 0.26	78 (5)
BDE-28	0.742 ± 0.027	0.467 ± 0.067	63 (5)
BDE-47	29.9 ± 2.3	30.2 ± 5.1	101 (5)
BDE-99	18.5 ± 2.1	22.0 ± 3.7	119 (16)
BDE-100	8.57 ± 0.52	9.04 ± 1.8	105 (9)
BDE-153	2.81 ± 0.41	3.16 ± 0.69	112 (9)
BDE-154	5.77 ± 0.80	6.57 ± 1.2	114 (12)

^a^ Mean value ± expanded combined measurement uncertainty (*U_r,tot_*); *n* = 3.

**Table 6:** Summary of uncertainties obtained for the test analytes using the top-down approach.

Analyte	*u_r,repro_* (%)	*B_r_* (%)	*u_r,cm_* (%)	*u_r,ref_* (%)	*u_r,tot_* (%)	*U_r,tot_* (%)
Hexachloro-1,3-butadiene	8.01	−4.66	0.909	1.15	9.38	18.8
Pentachlorobenzene	9.90	−5.15	0.676	0.250	11.2	22.4
*alpha*-HCH	7.83	−10.6	0.154	0.475	13.2	26.3
Hexachlorobenzene	8.42	7.38	0.274	1.00	11.2	22.5
*beta*-HCH	8.13	−11.6	0.954	0.150	14.2	28.4
Lindane	6.04	−13.0	0.451	0.866	14.4	28.7
*delta*-HCH	7.15	−9.40	1.34	0.330	11.9	23.8
Heptachlor	7.60	−6.23	2.74	0.250	10.2	20.4
BDE-28	6.48	−1.28	2.78	0.295	7.18	14.4
BDE-47	7.98	1.90	1.56	0.300	8.36	16.7
BDE-100	9.75	0.192	0.913	0.300	9.80	19.6
BDE-99	8.01	−0.879	2.06	0.300	8.32	16.6
BDE-154	8.50	1.17	2.89	0.300	9.06	18.1
BDE-153	9.00	5.08	3.50	0.300	10.9	21.8

Abbreviations: *u_r,repro_*—within-lab reproducibility; *B_r_*—relative bias; *u_r,cm_*—uncertainty of systematic error; *u_r,ref_*—uncertainty of purity of analytical standard; *u_r,tot_*—relative combined measurement uncertainty; *U_r,tot_*—expanded combined measurement uncertainty.

**Table 7:** Analysis of samples of different fish species.

Analyte	European chub Concentr. ^a^/*RR* ^b^ (µg/kg/%)	Crucian carp Concentr. ^a^/*RR* ^b^ (µg/kg/%)	European perch Concentr. ^a^/*RR* ^b^ (µg/kg/%)	Northern pike Concentr. ^a^/*RR* ^b^ (µg/kg/%)	Zander Concentr. ^a^/*RR* ^b^ (µg/kg/%)	Brown trout Concentr. ^a^/*RR* ^b^ (µg/kg/%)	Atlantic salmon Concentr. ^a^/*RR* ^b^ (µg/kg/%)	Alaska pollock Concentr. ^a^/*RR* ^b^ (µg/kg/%)
Hexachloro-1,3-butadiene	<0.09/96 (2)	<0.09/86 (6)	<0.09/87 (1)	<0.09/95 (1)	<0.09/93 (1)	<0.09/116 (1)	0.90 ± 0.01/98 (4)	0.22 ± 0.01/104 (3)
Pentachlorobenzene	<0.12/92 (3)	<0.12/96 (6)	<0.12/90 (2)	<0.12/105 (4)	<0.12/87 (3)	<0.12/108 (3)	0.22 ± 0.01/95 (1)	<0.12/104 (3)
*alpha*-HCH	<0.10/85 (2)	<0.10/98 (8)	<0.10/84 (2)	<0.10/92 (8)	<0.10/83 (6)	<0.10/105 (4)	0.23 ± 0.01/86 (3)	<0.10/95 (2)
Hexachlorobenzene	1.00 ± 0.01/95 (2)	0.35 ± 0.01/92 (3)	0.48 ± 0.01/93 (1)	1.84 ± 0.02/96 (3)	0.70 ± 0.01/95 (2)	1.12 ± 0.01/118 (2)	2.68 ± 0.05/96 (4)	0.18 ± 0.01/99 (1)
*beta*-HCH	<0.12/82 (3)	<0.12/93 (8)	<0.12/82 (2)	<0.12/97 (11)	<0.12/76 (7)	0.80 ± 0.02/113 (7)	0.12 ± 0.01/81 (4)	<0.12/93 (3)
Lindane	<0.13/83 (3)	<0.13/93 (8)	<0.13/82 (2)	<0.13/91 (9)	<0.13/79 (6)	<0.13/101 (6)	<0.13/84 (4)	<0.13/91 (2)
*delta*-HCH	<0.12/82 (2)	<0.12/92 (8)	<0.12/82 (3)	<0.12/102 (10)	<0.12/78 (7)	0.13 ± 0.003/100 (7)	<0.12/82 (4)	<0.12/90 (3)
Heptachlor	<0.13/78 (6)	<0.13/64 (12)	<0.13/98 (3)	<0.13/71 (6)	<0.13/114 (8)	<0.13/106 (8)	<0.13/83 (4)	<0.13/65 (10)
BDE-28	<0.12/89 (7)	<0.12/128 (9)	<0.12/88 (2)	<0.12/110 (5)	<0.12/99 (8)	<0.12/87 (4)	<0.12/86 (4)	<0.12/114 (9)
BDE-47	0.69 ± 0.01/95 (2)	<0.09/100 (2)	1.33 ± 0.04/103 (5)	0.21 ± 0.01/104 (6)	1.45 ± 0.01/103 (3)	0.36 ± 0.01/94 (4)	0.43 ± 0.02/97 (5)	<0.09/94 (0.4)
BDE-100	0.20 ± 0.002/100 (9)	<0.09/94 (8)	0.26 ± 0.02/111 (5)	<0.09/105 (9)	0.18 ± 0.01/96 (6)	0.10 ± 0.01/97 (6)	0.09 ± 0.01/105 (10)	<0.09/71 (12)
BDE-99	<0.07/96 (9)	<0.07/91 (8)	0.41 ± 0.02/113 (6)	<0.07/111 (14)	<0.07/96 (6)	0.25 ± 0.01/94 (5)	<0.07/104 (9)	<0.07/67 (4)
BDE-154	<0.16/103 (10)	<0.16/100 (21)	<0.16/120 (3)	<0.16/128 (15)	<0.16/99 (9)	<0.16/97 (7)	<0.16/112 (13)	<0.16/53 (15)
BDE-153	<0.14/99 (14)	<0.14/103 (23)	<0.14/119 (3)	<0.14/114 (19)	<0.14/95 (7)	<0.14/95 (6)	<0.14/100 (2)	<0.14/53 (16)
Lipid content (%)	3.5	0.96	3.0	2.4	1.5	8.2	16	0.63
Moisture content (%)	78	74	74	78	77	71	58	81

^a^ For positive samples: mean value ± SD; *n* = 3. For negative samples: <LOQ value. ^b^ *RR*—relative recovery; in parenthesis: RSD value; *n* = 5.

**Table 8:** Analytical Eco-Scale assessment of the proposed method according to Galuszka et al. [31].

	Penalty Points
*Reagents*	
MeCN (5 mL)	4
CHCl_3_ (50 µL)	2
Hexane (80 µL)	8
Analytes standard solution	4
H_2_O (4 mL)	0
H_2_SO_4_ (1 mL)	2
MgSO_4_ (2 g)	0
NaCl (0.5 g)	0
CH_3_COONa	0
*Instruments*	
Vortex	1
Centrifuge	1
GC–MS/MS	3
*Occupational hazard*	3
*Waste*	4
*Total penalty points*	Σ 32
*Analytical Eco-Scale total score*	68

**Table 9:** Comparison of the developed method with other QuEChERS based methods with enhanced sample extract clean-up for determination of test analytes in fish.

Analytes	Extractant	Clean-Up	Analysis	Recoveries (%)	LOQs (µg/kg)	Reference
Pesticides	MeCN	Dual dSPE (1. PSA + C18 + MgSO_4_; 2. PSA + C18 + MgSO_4_)	GC–ECD	57–98	1.5–3.5	[9]
Pesticides	MeCN or MeCN/THF (3:1)	Freezing (2 h), dual dSPE (1. CaCl_2_; 2. PSA + MgSO_4_)	GC–MS	43–113	1–10	[12]
Pesticides	MeCN + CHCl_3_ (10:1)	Dual dSPE (1. PSA + SAX +NH_2_ + MgSO_4_; 2. C18), freezing (overnight)	GC–MS	61–102	4–6	[13]
Pesticides	MeCN + hexane (15:2)	Freezing (20 min), dual dSPE (1. CaCl_2_ + MgSO_4_; 2. PSA + florisil + C18 +MgSO_4_)	GC–MS/MS	60–127	2–13	[14]
PBDEs	MeCN (sonication)	Dual dSPE (1. PSA + C18 + MgSO_4_; 2. PSA + C18 + MgSO_4_)	GC–MS	60–107	<15	[15]
PBDEs	Ethyl acetate	GPC, SPE (silica + Na_2_SO_4_)	GC–MS	88–140	0.09–2.2	[16]
Pesticides	MeCN	Freezing (min. 4 h), dSPE (Z-Sep + MgSO_4_), filtration (0.2 µm PTFE filter)	GC–MS/MS	86–101	0.08–0.15	[32]
PBDEs	MeCN + toluene (4:1)	Dual dSPE (1. EMR-Lipid; 2. Z-Sep + MgSO_4_)	GC–MS/MS	79–116 (muscle) 89–107 (liver)	0.015–0.065 0.85–1.1	[33]
Pesticides, PBDEs	MeCN	pH-tuned DLLME (0.5 M CH_3_COONa, CHCl_3_), H_2_SO_4_ clean-up	GC–MS/MS	57–124 (pesticides) 93–110 (PBDEs)	0.09–0.17 0.07–0.16	This work

Abbreviations: MeCN—acetonitrile; dSPE—dispersive solid-phase extraction; PSA—primary secondary amine; C18—octadecyl silica; GC—gas chromatography; ECD— electron-capture detector; THF—tetrahydrofurane; MS—mass spectrometry; SAX—strong anion exchange resin; GPC—gel permeation chromatography; SPE—solid-phase extraction cartridge; Z-Sep, EMR-Lipid—clean-up sorbents; PTFE—polytetrafluoroethylene (Teflon); DLLME— dispersive liquid–liquid microextraction.
